# Metabolic Syndrome Spectrum in Children with Classic Congenital Adrenal Hyperplasia—A Comprehensive Review

**DOI:** 10.3390/metabo15020089

**Published:** 2025-02-02

**Authors:** Sanja Panic Zaric, Tatjana Milenkovic, Sladjana Todorovic, Katarina Mitrovic, Dimitrije Cvetkovic, Maja Cehic, Jelena Vekic, Katja Dumic, Rade Vukovic

**Affiliations:** 1 Department of Pediatric Endocrinology, Mother and Child Health Care Institute of Serbia “Dr Vukan Cupic”, 11 070 Belgrade, Serbia; 2 School of Medicine, University of Belgrade, 11 000 Belgrade, Serbia; 3 Department of Medical Biochemistry, Faculty of Pharmacy, University of Belgrade, 11 221 Belgrade, Serbia; 4 Department of Pediatric Endocrinology and Diabetes, University Hospital Centre Zagreb, University of Zagreb School of Medicine, 10 000 Zagreb, Croatia

**Keywords:** classical congenital adrenal hyperplasia, children, metabolic syndrome, metabolic health, cardiometabolic, cardiovascular, hypertension, obesity, insulin resistance, dyslipidemia

## Abstract

Children with a classic form of congenital adrenal hyperplasia (CCAH) have a potentially increased risk of unfavorable cardiometabolic events due to the interplay of corticosteroid treatment, hyperandrogenism, and other factors. Although readily recognized in adults, these aspects are frequently overlooked in children and youth with CCAH; Aim: To review the evidence available from studies regarding cardiometabolic health outcomes in CCAH patients; Methods: A review of the literature was performed following PRISMA guidelines, including studies published between 2000 and 2024. We included studies reporting cardiometabolic outcomes in children and adolescents (<18 years) with CCAH. Where pediatric data were sparse, additional data were obtained from studies with older adolescents and young adults (15–25 years). Cardiometabolic outcomes included risk factors, such as obesity, insulin resistance, lipids, blood pressure, and vascular markers; Results: Twenty-five studies were analyzed. The prevalence of obesity was found to be higher in children with CCAH, as well as of increased visceral adiposity. Higher indices of insulin resistance were also a frequent finding in children with CCAH. CCAH patients had higher systolic blood pressure and more frequently loss of nocturnal blood pressure dipping, particularly among salt-wasting subtypes and in younger children. Subclinical atherosclerosis was indicated by increased carotid intima–media thickness, elevated hs-CRP, and impaired endothelial function. Other findings suggested changes in lipid profiles, particularly decreased HDL-c and increased triglycerides, although the findings were less consistent; Conclusions: Compared with the general pediatric population, children with CCAH were found to have an increase in multiple cardiometabolic risk factors. It is therefore vital to monitor these risk factors in pediatric CCAH, as well as tailoring treatment with cardiometabolic health in mind, to achieve better long-term cardiovascular and metabolic outcomes. Future research should focus on longitudinal studies of cardiometabolic outcomes and innovative therapeutic approaches to reduce these risks in patients with CCAH.

## 1. Introduction

Congenital adrenal hyperplasia (CAH) caused by 21-hydroxylase deficiency (21OHD), resulting from mutations in the *CYP21A2* gene, is an autosomal recessive condition that requires lifelong treatment and impacts the quality of life [1]. These children have cortisol and often aldosterone deficiency, combined with overproduction of adrenal androgens due to chronically elevated ACTH levels [2]. CAH, due to 21OHD, includes two forms: classic and non-classic [3]. The classic form is further defined as either the salt-wasting (SW), which presents in the neonatal period due to severe deficiency of both cortisol and aldosterone, or the simple-virilizing (SV) form, which usually presents with mostly cortisol deficiency. Both the SW and SV forms also present with androgen excess, leading to various degrees of external genitalia virilization in affected females and peripheral precocious puberty in both sexes [4]. The non-classic form is the mildest form of the disease, which presents later in childhood with varying degrees of symptoms attributed to hyperandrogenemia [5].

The goals of medical treatment are to substitute for the lack of glucocorticoids (GCs) and mineralocorticoids (MCs), as well as to reduce the overproduction of adrenal androgens by suppressing ACTH oversecretion. This approach should result in the prevention of adrenal crises and the promotion of normal growth in childhood [6]. However, treatment typically requires supraphysiological doses of GCs in order to suppress ACTH and consequential androgen overproduction [7]. In clinical practice, treating classic CAH is striking a balance between overtreatment, which can lead to hypercortisolism, and undertreatment, which may result in androgen excess [6]. Apart from common long-term complications of under- or overtreatment, such as impaired growth and final height, both hypercortisolism and androgen excess have been linked to adverse cardiometabolic outcomes, including metabolic syndrome components [7,8,9,10,11].

Previously, these risks of the treatment of pediatric CAH have not received much attention, as they typically do not become apparent until later in adulthood. However, there is growing evidence that early stages of atherosclerosis begin to develop already during childhood, especially in high-risk populations, such as children with classic CAH caused by 21OHD (CCAH). This highlights the need to focus our attention on non-traditional cardiometabolic outcomes in pediatric patients with CCAH [6]. Patients with CCAH experience an increased cardiometabolic risk compared to the general population associated with obesity, insulin resistance (IR), hypertension (HTA), and dyslipidemia, leading to premature cardiovascular disease (CVD) and metabolic syndrome (MetS) [12]. Although the full extent of pathogenic processes leading to these metabolic effects is yet to be defined, the bulk of the added cardiometabolic risk is believed to be due to the effects of supraphysiological GC therapy and/or androgen excess [12]. Even though current and advancing treatment methods have led to decreased mortality rates and enhanced quality of life for patients, they still face ongoing challenges from lifelong metabolic and cardiovascular conditions such as obesity, type 2 diabetes, hyperlipidemia, and HTA, all of which negatively impact their longevity and overall quality of life [13].

This study aimed to review clinical research evidence on cardiometabolic health outcomes in children with CCAH due to 21OHD. It highlights factors contributing to increased CVD risk, including obesity, body composition, hypertension, glucose metabolism, insulin resistance, and dyslipidemia.

## 2. Methods

The inclusion criteria involved observational, longitudinal, cross-sectional, and case–control studies published within the period from 2000 to 2024, evaluating outcomes related to cardiometabolic health in children and adolescents with CCAH due to 21OHD. We obtained all the data (specified in detail below, in the “Independent variables”, “Outcome variables” section of the Method) from the studies in age group less than 18 years. For the cardiometabolic risk factors for which data were scarce in the population <18 years, we obtained data from the adolescent/young adult age group up to 25 years. Only studies published in English were included in the analysis.

The following are the exclusion criteria: the full text was not possible to obtain, studies with other forms of CAH (non-classic CAH, mixed 11 beta-hydroxysteroid deficiency and CCAH as well mixed CCAH and non-classic CAH), studies in which the type of CAH was not explicitly defined, studies in adults with CAH, and studies conducted before 2000.

### 2.1. Study Identification and Search Strategy

**Figure 1:**
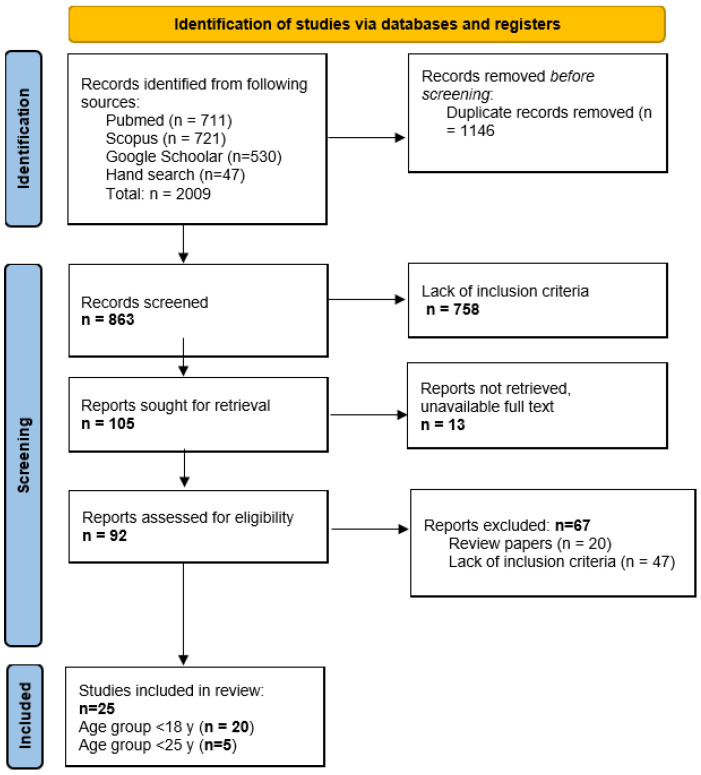
PRISMA flowchart of the review process.

The present research was designed and performed as a review. The search involved the PubMed, Scopus, and Google Scholar databases. In addition to the electronic database search, we also conducted a manual citation search to identify all relevant studies. This involved a parallel-process search strategy to locate studies, involving both backward citation searching in the form of reviewing reference lists of all included articles, and forward citation searching in the form of tracking included studies through the citation database (Google Scholar) for related studies citing these articles. The search was performed in February 2024 using a combination of keywords: “congenital adrenal hyperplasia”, “metabolic syndrome”, “metabolic health”, “cardiometabolic”, “cardiovascular”, “acute myocardial infarction”, “coronary heart disease”, “stroke”, “hypertension”, “cardiovascular morbidity”, “cardiac disease”, “systolic and diastolic blood pressure”, “vascular endothelial dysfunction”, “heart failure”, “carotid intima media thickness”, “atrial fibrillation”, “venous thromboembolism”, “lipids”, “lipid profile”, “high triglyceride levels”, “low HDL cholesterol”, “hyperlipidemia”, “low HDL cholesterol”, “high total cholesterol”, “high LDL cholesterol”, “dyslipidemia”, “atherogenesis”, “atherosclerosis”, “atherogenic dyslipidemia”, “oxidative stress”, “homeostatic model assessment for insulin resistance”, “obesity”, “insulin resistance”, “diabetes type 2”, “hyperglycemia”, “overweight”, “impaired fasting blood glucose”, “body mass index”, “metabolic morbidity”, “metabolic syndrome”, “metabolic disease”, “HBA1c”, “redox status”, “antioxidative defense”, “prooxidants”, “lipid status”, “lipid parameters”, “small dense LDL”, “LDL subclasses”, “lipid profile”. The study identification, search strategy, and reporting of this review followed the Preferred Reporting Items for Systematic Reviews (PRISMA) guideline (Figure 1) [14].

### 2.2. Study Selection and Data Collection

Two authors (S.P.Z. and R.V.) independently evaluated all retrieved abstracts, selected full-text manuscripts for eligibility, extracted all data and, if needed, standardized or comparative purposes. A database of all identified studies was created using MS Excel 2010. After the screening, retrieval, and eligibility assessment, a total of 25 studies were included in the final analysis (Figure 1).

### 2.3. Independent Variables

For each study, standard clinical characteristics of the included population were documented, such as age; gender; anthropometric features (body height, body weight); CCAH type (SW or SV); serum androgen levels (testosterone, 17OHP).

### 2.4. Outcome Variables

The outcomes of interest were measures of obesity (body mass index, waist circumference, waist-to-height ratio, waist-to-hip ratio) and body composition (triceps and subscapular skinfold thickness, dual-energy x-ray absorptiometry analysis, visceral adipose tissue (VAT), subcutaneous adipose tissue (SAT)), IR and glucose metabolism (HOMA IR, OGTT), dyslipidemia (triglycerides, total cholesterol, high-density lipoprotein, low-density lipoprotein, lipoprotein subclasses), HTA (systolic blood pressure, diastolic blood pressure, physiological nocturnal dip in systolic blood pressure) and other cardiometabolic risk factors: carotid intima–media thickness, endothelial function, inflammatory markers, and oxidative stress parameters (Table 1).

### 2.5. Risk of Bias

After completion of data extraction, one author (K.D.) randomly selected 10% of the papers and checked each data entry field to assess whether data extraction was carried out correctly. Inter-researcher agreement was 97.19% (Kappa score 0.82). Disagreements during the screening process were resolved through consensus.

## 3. Pathophysiological Mechanisms

The primary objectives of GC treatment are to replace deficient cortisol and suppress the overproduction of androgens while aiming to reduce the occurrence of Cushingoid symptoms [15,35]. Glucocorticoid regimens are also individualized to compensate for stress and to suppress ACTH sufficiently to maintain adrenal androgen excess under control. In order to do so, supraphysiological dosages of GCs are often required [36]. Although new formulations and new drugs are constantly under development, to date, no GC formulation has yet been able to replicate the physiological circadian rhythm of cortisol secretion accurately [9]. Due to the narrow therapeutic window of GC treatment, the prevailing side effects of both hyperandrogenism and hypercortisolism lead to additional metabolic and cardiovascular (CV) adverse outcomes [9]. Merke et al., in a phase 3 study, compared treatment with modified-release hydrocortisone (MR-HC) vs. standard GC and showed improved biochemical disease control in adults with a reduction in steroid dose over time and patient-reported benefits [37]. Jones et al. indicated that MR-HC reduced 17OHP and alternative pathway metabolite excretion to near-normal levels more consistently than other GC preparations [38]. Also, Sarafoglou et al., in phase 3 trial of corticotropin-releasing factor type 1 receptor antagonist (Crinecerfont) in pediatric patients with CCAH, reported that Crinecerfont was superior to a placebo in reducing elevated androstenedione levels and was also associated with a decrease in the GC dose from supraphysiologic to physiologic levels while androstenedione control was maintained [39]. Hopefully, further progress and improvement of new medications for CCAH should aim to reduce metabolic adverse outcomes, especially if these metabolic outcomes are taken into account during the development of new therapies.

**Figure 2:**
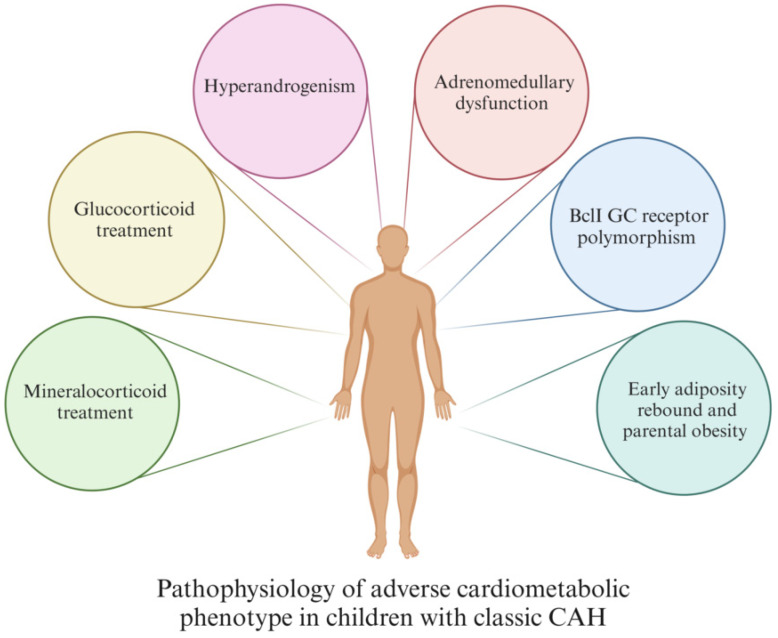
Pathophysiological mechanisms in children with CCAH.

Although numerous factors might be involved in the pathophysiology of CV risk factors development in children with CCAH, the following are considered to play a major role (Figure 2):

1. GC treatment
2. MC treatment
3. Hyperandrogenism
4. Adrenomedullary dysfunction
5. BclI GC receptor polymorphism
6. Early adiposity rebound and parental obesity

1. *GC treatment*: The therapeutic range of GC is tight, and precise and individualized follow-ups are needed for patients to avoid both under- and over-treatment, as both can increase the CV risk in CCAH patients [35,40,41,42,43,44]. GC treatment has been shown to be strongly correlated with obesity [40,41,42,43], HTA [41,42], impaired insulin sensitivity [8,40,41], and increased CV mortality [40].
2. *MC treatment*: Mineralocorticoid treatment increases blood pressure (BP) by acting on the MC receptor [8]; hence, management with excessively high doses of MC may result in high BP [35,36,40,41,42,44]. Therefore, it is important to individualize MC therapy based on BP, growth, and electrolyte values, as a precise dosing of MC treatment would minimize the risk of overtreatment [8,40,42].
3. *Hyperandrogenism*: Many CCAH patients have a certain degree of hyperandrogenism, even when they are receiving regular treatment. Androgen excess is a well-known CV risk factor [45,46,47]. In addition, excess androgens are linked to reduced insulin sensitivity, which is also a significant CV risk factor [40,41,43]. Insulin further promotes adrenal and ovarian steroidogenesis and acts in a positive feedback loop as a major driving pathophysiologic mechanism behind hyperandrogenism [48].
4. *Adrenomedullary dysfunction*: Patients with CCAH experience adrenomedullary dysfunction leading to reduced release of catecholamines, such as adrenaline, which normally facilitates lipolysis and suppresses insulin secretion via adrenergic receptors, thereby preventing a surge in fat mass [41,43,48,49]. This could be explained as the consequence of antenatal adrenomedullary maldevelopment due to decreased intra-adrenal GC [35,41,48]. Additionally, prolonged adrenomedullary dysfunction may also lead to increased insulin levels and IR [48].
5. *BclI GC receptor polymorphism*: Classic CAH patients carrying BclI variants of the GC receptor gene, which enhances the receptor’s transactivation process, are at increased risk of systolic hypertension and higher BMI and WC compared to the wild-type CCAH controls [50].
6. *Early adiposity rebound and parental obesity*: Early adiposity rebound (AR) is a well-known risk factor for childhood obesity and MetS [36,40], with potentially greater significance in children with CCAH, because in these children, AR has been found to occur at an earlier age than usual (at 1.7 years of age in the UK, 3 years of age in Japan, and 3.3–3.8 years of age in the USA) [16].

## 4. Obesity and Body Composition

Increased fat mass and abdominal adiposity are key risk factors in the evolution of CV diseases [41]. Out of twenty-five selected studies regarding cardiometabolic risk in CCAH children, nutritional status was assessed using the BMI in twenty-four, while eight studies also assessed waist circumference (WC), three used the waist-to-height ratio, and two evaluated the waist-to-hip ratio.

Of the twenty-four studies where BMI was evaluated, fifteen included a control group. In 8/15 studies, patients with CCAH had higher BMI compared to controls [4,10,17,18,19,20,21,22]. In five studies, no significant difference in BMI was found [6,7,15,23,24]. In one study, the control group consisted of age-matched obese children, while the CCAH group included 60% who were overweight and obese [25]. In a retrospective cross-sectional study, Subbarayan et al. found that 23.6% of the patients in the cohort were obese [26]. In the whole CCAH group, weight SDS and BMI SDS were significantly higher in both sexes when compared with the UK population mean [26,27]. A few studies reported a BMI greater than 2.0 SDS, suggesting a markedly higher rate of obesity in CCAH patients than expected for the normal population [28,29]. A high prevalence of overweight individuals in the CCAH group has been observed in other studies as well [3,15,16,20,23,27,28]. Studies showed that BMI SDS had a significant positive correlation with systolic and diastolic BP and IR [10,15], as well as a correlation to fludrocortisone and hydrocortisone doses [26,30]. The BMI SDS did not become notably higher with age, suggesting that, in CCAH, fat accumulation begins early in life [27]. In a study where BMI_HA_ percentile was calculated, fewer children were identified as height–age-obese compared to when they were assessed with regular BMI; however, the frequency of obesity was still high [31].

In eight studies that assessed WC [4,6,15,16,17,18,20,23], the majority (seven) had control groups. Only one study reported that increased WC was more prevalent in the control group [15]. Several studies indicated that there was no statistically significant difference in the waist-to-hip ratio between CCAH and healthy controls [6,16,17]. Other studies reported increased waist and hip circumference in the CCAH group [4,18,23]. Bacila et al. reported that the waist circumference SDS was higher in female CCAH patients and patients over 12 years compared to controls, while hip circumference was higher in the CCAH group only for males aged 8–12 years [20].

Out of all twenty-five selected studies, body composition was analyzed in four studies [16,18,23,27], with a control group in two of these studies [18,23]. One study evaluated triceps and subscapular skinfold thickness and detected higher skinfold thickness than expected for the UK population [27]. Two studies used dual-energy X-ray absorptiometry analyses (DXAs) [16,18]. In one of those studies, higher values of total body fat were observed in CCAH patients, compared to the control group [18]. Another retrospective study compared CCAH males and females and found that females have higher total body fat mass, body fat percentage, and trunk fat mass using the DXA, as well as higher VAT and VAT:SAT ratio using MRI [16]. In a cross-sectional study, Kim et al. observed profoundly raised abdominal adiposity (VAT, SAT) in CCAH compared with controls; the VAT:SAT ratio was also notably elevated in the CCAH and BMI z-score and the waist-to-height ratio; the trunk and total body fat mass correlated positively with the VAT and SAT [23]. In one study, which compared the body composition of children with CCAH with the control group, children with CCAH exhibited increased visceral adiposity [23].

In summary, the majority of the studies found a higher prevalence of obesity in children with CCAH when assessed using BMI and WC. Additionally, a few studies that assessed body composition in these children indicated an unfavorable body composition, showing higher levels of total fat and VAT compared to control groups.

## 5. Insulin Resistance and Glucose Metabolism

Insulin resistance is one of the driving forces behind MetS and a primary component of the unfavorable metabolic phenotype. Out of twenty-five selected studies regarding cardiometabolic risk in CCAH children, IR was assessed in sixteen, using HOMA-IR in all, while an oral glucose tolerance test (OGTT) was performed in two studies. Of the sixteen studies where IR was evaluated, fourteen included a control group. In 8/16 studies, CCAH patients had higher HOMA-IR compared to the controls [4,6,10,15,18,19,21,24]. In five studies, HOMA-IR did not differ from the controls [7,17,20,22,25]; although, in one of these studies, the controls were obese [25]. Two studies showed that HOMA-IR in patients with CCAH was positively related to age and was lower than the non-CAH individuals [22,26]. In one case–control study, Amr et al. reported that the median HOMA-IR and all median glucose levels, including fasting and glucose levels, measured at 30, 60, 90, and 120 minutes during OGTT, were significantly higher in the CCAH patients. Specifically, 34% of CCAH patients exhibited impaired fasting glucose, 19% had impaired glucose tolerance, and 34% had HOMA-IR higher than 2.7 and reduced insulin sensitivity compared to their healthy counterparts [19]. Similarly, in another case–control study conducted by Zhang et al., it was found that fasting-insulin concentrations and 2 h post-load plasma glucose levels were notably higher in patients with CCAH. Additionally, HOMA-IR was elevated, while the insulin sensitivity index was lower in these patients [24].

In summary, most of the studies found a higher prevalence of IR in children with CCAH compared to controls, mainly assessed using HOMA-IR.

## 6. Lipid Metabolism

The alterations in lipid metabolism that develop in childhood and adolescence are important risk factors for CV disease [32]. Among the twenty-five analyzed studies, the lipid profile (triglycerides, total cholesterol, high-density lipoprotein, low-density lipoprotein) was assessed in nineteen. None of the studies assessed lipoprotein subclasses. Of the nineteen studies in which the lipid profile was evaluated, fifteen included a control group. In 7/15 studies, the lipid profile was similar in children with CCAH compared to controls [6,7,10,17,18,19,51]. Five studies reported lower HDL-c levels in the CCAH group [4,15,20,22,24]. Two studies indicated higher LDL in the CCAH group compared with obese controls [25] and with healthy controls [4]. Three studies reported higher triglyceride (TG) levels in the CCAH group compared to healthy controls [4,21,24]. One study revealed significantly higher total cholesterol (TC) levels in CCAH patients compared with healthy controls [4]. Botero et al., in a case–control study, demonstrated that the percentage of CCAH patients who had TC and LDL-c levels exceeding the cutoff points was not statistically significant. However, there was a significant difference in serum TG levels, attributed to a higher number of individuals with abnormally elevated levels of TC, TG, and LDL-c in the group receiving GC. Therefore, the authors suggested that GC therapy may induce alterations in the lipid profile [33]. Two studies showed no significant correlations between mean daily hydrocortisone dose and lipid profile [19,32]. One study showed that the SW patients exhibited a less favorable lipid profile comparing with the SV patients, characterized by increased LDL-c, TG levels, and low HDL-c levels [15].

In summary, findings regarding lipid alterations in children with CCAH were inconsistent, with almost half of the studies indicating no changes to the lipid profiles of children with CCAH, and the other half reporting different alterations of lipid metabolism (lower HDL-c, higher LDL, higher TG and TC).

## 7. Hypertension

Childhood HTA poses a serious risk for CV disease later in life. The estimated prevalence of HTA in CCAH children is higher than the prevalence reported in the general pediatric population [42]. Out of the twenty-five selected studies assessing cardiometabolic risk in CCAH children, the BP was assessed in sixteen, in the majority (thirteen) using several single measurements, and in three studies using 24-hour ambulatory blood pressure monitoring (ABPM).

Of the sixteen studies where BP was evaluated, nine included a control group. Four studies indicated higher systolic blood pressure (SBP) in the CCAH group when compared to healthy controls [4,6,7,22]. Two studies compared obese controls with the CCAH group without obesity and found higher SBP in the obese controls [6,25]. Three studies showed that the majority of CCAH patients had an absence of the physiological nocturnal dip in SBP [17,27,30]. Two studies showed no difference between CCAH patients and the control group regarding SBP [17,18] or diastolic blood pressure (DBP) [7,18]. Two studies reported a higher DBP in CCAH group [4,17]. In a longitudinal study, Sarafoglou et al. demonstrated that the highest rate of HTA occurs in SW patients, particularly among males, starting before the age of five. In contrast, most cases of HTA in SV patients were observed after the age of five. The incidence rates of high BP remained above 50% for both SW and SV CCAH patients between the ages of ten and eighteen [34]. In cross-sectional and controlled study, Marra et al. presented that CCAH children exhibit elevated SBP responses during exercise, and both males and females shared a typical model of exercise reduction [18].

Bonfig et al. analyzed BP data of children and adolescents with CCAH and adjusted it for height, then compared it to contemporary German national reference data, and the results indicated that the overall prevalence of HTA was 12.5%, higher in younger children (age range 3–8 years) than in adolescents [30]. According to Bonfig et al., SW patients had significantly higher BP compared to SV patients. In the same study, BP measurements correlated significantly with MC dose in both age groups; up eight years of age and at the pubertal age (12–18 years), whereas HTA was more prevalent in females [30]. On the other hand, Ozdemir et al. did not find a correlation between BP and MC therapy [7]. Also, Kim et al. did not find a difference in MC doses between hypertensive and non-hypertensive patients and demonstrated that 75% of CCAH patients younger than 18 years old were normotensive, 11% were prehypertensive, and 14% were hypertensive [23]. Similar results were shown in a retrospective cross-sectional study with a study population within the age range of 0.4–20.5 years, where almost 21% of CCAH patients had systolic HTA, and 11% were prehypertensive; the mean SBP and SBP SDS were significantly elevated compared to the reference population [26].

In a retrospective case–control study regarding ABPM, the data revealed that 32% of CCAH children were hypertensive using casual BP readings, but 76% of patients experienced normal BP when classified by ABPM, 24% had daytime or nighttime HTA, 20% had impaired SBP, 16% had DBP dipping, and there were no important distinctions between the hypertensive and non-hypertensive CCAH group regarding anthropometric and metabolic parameters [17]. In a cross-sectional study regarding ABPM, Roche et al. reported that 58% of CCAH children (67% male, 52% female) had systolic HTA, 24% had diastolic HTA, and the mean SBP SDS was significantly higher than the BP of the reference population [27]. Volkl et al., in a prospective cross-sectional study regarding ABPM, showed that almost 11% of CCAH children had a mean daytime SBP above the 97th percentile, 80% above the 50th percentile, with notably reduced daytime DBP levels, while nighttime DBP was normal. The ABPM demonstrated higher SBP levels only in girls, while the DBP levels were reduced in boys [28].

In summary, the majority of the analyzed studies showed that HTA is more prevalent in CCAH patients compared to the general pediatric population, with SBP frequently elevated, especially in the SW group and in the age group of 3–8 years. The majority of CCAH patients also have a loss of nocturnal reduction in SBP. While therapy may impact BP, data are scarce and discrepant in this regard.

## 8. Other Cardiovascular Risk Factors

Apart from classical components of the MetS, some studies also analyzed the presence of additional CV risk factors in children with CCAH. Endothelial dysfunction is an initial sign of the development of atherosclerosis [12]. The carotid intima–media thickness (cIMT) serves as a screening tool for detecting subclinical atherosclerosis and acts as an independent predictor of future CV events [19]. High-sensitivity C-reactive protein (hs-CRP) has been utilized as a biomarker to assess disease prognosis in patients with CV risk, and elevated hs-CRP is a reputable indicator of microvascular dysfunction [52]. Oxidative stress parameters play a significant role in the development of atherosclerosis and CVD [53].

Out of the twenty-five selected studies examining cardiometabolic risk in CCAH children, cIMT was assessed in seven, hs-CRP in four studies, and endothelial dysfunction in one. Notably, all studies evaluating additional cardiovascular risk factors included a control group. The majority (six) of these studies reported significantly higher cIMT values in the CCAH group compared to controls [4,7,17,19,22,25]. Also, cIMT was higher in CCAH patients with nocturnal hypertension [17,25]. One study found no significant differences in cIMT between CCAH patients and the control group or any differences between obese and CCAH patients. Regarding hs-CRP, studies showed higher hs-CRP in the CCAH group [4,6] in obese controls [6] and also in uncontrolled CCAH patients [4]. Interestingly, prepubertal CCAH patients exhibited significantly higher levels of hs-CRP compared to healthy controls. However, there was no significant difference in hs-CRP levels between the pubertal subgroups of CAH patients and the healthy controls [21]. In assessing endothelial function through the evaluation of flow-mediated dilatation and smooth muscle function glyceryl tri-nitrate dilatation, data indicated that CCAH children who are not obese had significantly reduced endothelial function. This impairment was found to be similar in obese control subjects [6]. In comparison to healthy controls, the CCAH group exhibited increased epicardial fat thickness, left ventricle mass index, indicating myocardial hypertrophy, and prolonged mitral deceleration time, which signifies compromised diastolic function [4].

In summary, children with CCAH were found to have significantly higher cIMT values and elevated hs-CRP levels. Although data are scarce, findings also indicate impaired endothelial function compared to controls. None of the studies did report oxidative stress parameters.

## 9. Discussion

This review summarizes the findings of available studies on cardiometabolic risk in children with CCAH. Recently, awareness and understanding of the long-term risks of CCAH and its treatment have been raised. Although improvements in GC and MC therapies have led to better clinical outcomes and improved quality of life, increasing evidence suggests that adults in this population face a higher risk of developing health problems, which all have significant consequences for CVD and MetS. Studies show that CCAH adults suffer mostly from obesity, HTA, IR, and higher cIMT [8,11,40,41,43,54]. Also, the mortality rate appears to be higher in the CCAH group, which is particularly concerning [55]. Furthermore, ongoing research points out that CCAH children are also likely to experience metabolic alterations starting from early childhood, which increases their risk of developing CVD later in life [43,49]. Having this in mind, it is crucial to closely adjust medical supervision, patient monitoring, and treatment regimens during childhood.

The majority of the studies found a higher prevalence of obesity in children with CCAH, emphasizing the need for targeted prevention strategies for this population. Additionally, few studies that assessed body composition reported unfavorable body composition in children with CCAH. This indicates the necessity for further research on body composition and other measures of adiposity in this group of children with CCAH. Also, most of the studies reported higher prevalence of IR in children. One study showed a significant correlation between the plasma testosterone levels and HOMA-IR [24]. In contrast, another study reported no statistically significant correlation with androgen serum levels (17OHP, testosterone, DHEAS) and HOMA-IR in the CCAH group [3]. The data indicated that HOMA-IR positively correlated with VAT and SAT [23], hydrocortisone dose [25], and epicardial fat thickness [4]. This highlights the need for further studies to explore the relationship between IR and androgen levels in CCAH children as well as the necessity for appropriate treatment regimens.

With regard to dyslipidemia in children with CCAH, findings were inconsistent. Some studies showed no difference in the lipid profile of CCAH patients, while others reported various alterations of lipid metabolism (lower HDL-c, higher LDL, higher TG and TC). The findings concerning lipid metabolism are variable and limited in children with CCAH as well as in adults, and there are no data regarding lipoprotein subclasses; so, further research regarding lipid alterations in children with CCAH is needed. The majority of analyzed studies showed that HTA is more prevalent in CCAH patients compared to the general pediatric population, with SBP frequently elevated, especially in the SW group and in the age group of 3–8 years. Also, the majority of CCAH patients have a loss of nocturnal reduction in SBP. According to these findings, close monitoring, particularly in the SW group and the age group of 3–8 years, is needed because these patients are at a higher risk for elevated SBP, as well as BP assessments during regular check-ups for all CCAH children and lifestyle modifications, such as a healthy diet and physical activity. While therapy may influence BP, existing data are limited and inconsistent. Further research is crucial to fully understand the underlying mechanisms and to develop a personalized management strategy that effectively addresses both daytime and nighttime blood pressure regulation.

Children with CCAH have significantly higher cIMT values and elevated hs-CRP levels. Some findings also suggest impaired endothelial function; however, the data are insufficient, indicating that additional studies are necessary to profoundly understand these aspects. Further research should focus on the investigation of oxidative stress parameters and their role in CV risk among children with CCAH and exploring interventions that could improve endothelial function and mitigate long-term cardiovascular complications.

The limitation of the present study is that only studies published during the period from 2000 to 2024 and indexed in PubMed, Google Scholar, and Scopus databases were included in the analysis, leaving the possibility that other relevant results published outside of these specified limits were omitted from our analysis.

These data should lead to a re-evaluation of management paradigms for CCAH in children, specifically broadening the focus from adrenal crisis avoidance and growth outcomes to cardiometabolic health. Regular screening for obesity, IR, dyslipidemia, HTA, and early vascular dysfunction is essential to reduce long-term CV risk. Having this in mind, our suggestions for additional monitoring of children with CCAH who are transitioning to adulthood regarding CVD risk factors would be to perform the following assessments before the transition to adult care: OGTT, 24 h ABPM, cardiology evaluation with heart ultrasound and cIMT measurement, bioimpedance with DXA, and lipid status profile. To optimize medical care throughout a lifetime in this high-risk population, interdisciplinary collaboration between pediatric endocrinologists, adult endocrinologists, and cardiologists is required. Longitudinal studies should be prioritized to investigate the long-term clinical course of cardiometabolic outcomes in CCAH while exploring novel therapeutic approaches and studying biomarkers.

## Figures and Tables

**Table 1:** Overview of analyzed studies regarding cardiometabolic risk.

						Metabolic Factors Analyzed				
No	Autor	Year	Age Group	Study Population	Control Group	Obesity	Glucose Metabolism	Lipids	Blood Preassure	Other
**1**	Hashemi Dehkordi et al. [3]	2021	<18 y	78 patients (51 SW, 27 SV)	No control group	BMI	HOMA IR	TG, TC, LDL, HDL	single measurments	
**2**	Metwalley et al. [4]	2019	<18 y	36 patients (SW 30, SV 6)	36 healthy controls matched for age, gender, pubertal, socioeconomic status	BMI ˚, WC ˚	HOMA IR ˚	TG ˚, TC ˚, LDL ˚, HDL ˚	single measurments ˚	hsCRP ˚, cIMT ˚
**3**	Harrington et al. [6]	2012	<18 y	14 patients (3 SV, 11 SW)	28 obese controls and 53 healthy controls	BMI, WC	HOMA IR˚	TG, TC, HDL	single measurments ˚	hsCRP ˚, cIMT, endothelial function ˚
**4**	Özdemir et al. [7]	2016	<18 y	25 patients ˜	25 matched controls in terms of age, gender, and body size	BMI	HOMA IR	TG, TC, LDL, HDL	single measurments ˚	cIMT ˚
**5**	Vijayan et al. [10]	2019	<25 y	52 patients ˜	28 healthy age- and sex-matched controls	BMI ˚	HOMA IR ˚	TG, TC, HDL, LDL	single measurments	
**6**	Moreira et al. [15]	2013	<18 y	33 patients (11 SV, 22 SW)	33 controls matched on BMI, age and sex	BMI, WC	HOMA IR ˚	TG, TC, HDL ˚, LDL		
**7**	Bhullar et al. [16]	2020	<18 y	42 patients (38 SW, 4 SV)	No control group	BMI, WC, DXA, MRI		TG, HDL	single measurments	
**8**	Akyürek et al. [17]	2015	<18 y	25 SW patients	25 age- and sex-matched healthy controls with normal weight and height percentiles	BMI ˚, WC	HOMA IR	TG, TC, LDL, HDL	24 h ABPM ˚	cIMT ˚
**9**	Marra et al. [18]	2015	<18 y	20 patients (15 SW, 5 SV)	20 healthy adolescents, statistically not different for sex, pubertal status, and physical activity; 18 age- and BMI-matched patients affected by JIA	BMI ˚, WC ˚, DXA ˚	HOMA IR ˚	TG, TC, LDL, HDL	single measurments	
**10**	Amr et al. [19]	2014	<18 y	32 patients, (24 SW, 8 SV)	32 healthy controls	BMI ˚	HOMA IR ˚, OGTT ˚	TG, TC, LDL, HDL		cIMT ˚
**11**	Bacila et al. [20]	2022	<18 y	107 patients ˜	83 healthy age- and sex matched controls differed by ethnicity	BMI ˚, WC ˚	HOMA IR	TG, TC, HDL ˚, LDL	single measurments	
**12**	Kurnaz et al. [21]	2020	<18 y	56 patients, (36 SW, 20 SV)	70 age- and sex-matched healthy controls	BMI ˚	HOMA IR ˚	TG ˚, TC, HDL, LDL		hsCRP
**13**	Rodrigues et al. [22]	2015	<25 y	40 patients (29 SW, 11 SV)	73 healthy, normal-weight children and adolescents	BMI ˚	HOMA IR	TG, TC, HDL ˚, LDL	single measurments ˚	cIMT ˚
**14**	Kim et al. [23]	2015	<18 y	28 patients (20 SW, 8 SV)	28 healthy controls matched for age, sex, ethnicity, and BMI	BMI, WC measured from CT images ˚, VAT ˚, SAT ˚	HOMA IR	TG, TC, HDL, LDL, VLDL	single measurments	hsCRP
**15**	Zhang et al. [24]	2010	<25 y	30 patients (30 untreated SV women)	30 controls	BMI	HOMA IR ˚, OGTT ˚	TG ˚, TC, HDL ˚, LDL	single measurments	hsCRP
**16**	Abdel Meguid et al. [25]	2022	<18 y	30 patients ˜	66 age-matched obese children	BMI	HOMA IR	TG, TC, HDL, LDL ˚	single measurments	cIMT ˚
**17**	Subbarayan et al. [26]	2014	<25 y	107 patients (85 SW, 22 SV)	No control group	BMI ˚	HOMA IR	TG, TC	single measurments ˚	
**18**	Roche et al. [27]	2003	<18 y	38 SW patients	No control group	BMI ˚, triceps and subscapular skinfold thickness ˚			24 h ABPM ˚	
**19**	Völkl et al. [28]	2006	<25 y	55 patients SW 45, SV 10	No control group	BMI ˚			24 h ABPM	
**20**	Völkl et al. [29]	2015	<18 y	89 patients ˜	No control group	BMI ˚			single measurments	
**21**	Bonfig et al. [30]	2015	<18 y	716 patients (571 SW, 145 SV)	No control group	BMI			single measurments ˚	
**22**	Sarafoglou et al. [31]	2017	<18 y	194 patients (124 SW, 70 SV)	No control group	BMI				
**23**	Doerr et al. [32]	2020	<18 y	43 patients (37 SW, 6 SV)	No control group	BMI		TG, TC, HDL, LDL, non-HDL		
**24**	Botero et al. [33]	2000	<18 y	14 patients ˜	14 prepubertal children			TG, TC, HDL, LDL		
**25**	Maccabee-Ryaboy et al. [34]	2016	<18 y	180 patients (120 SW, 60 SV)	No control group	BMI			single measurments	

SW-salt wasting; SV-simple virilizing; BMI-body mass index; WC-waist circumference; VAT-visceral adipose tissue; SAT-subcutaneous adipose tissue; DXA-dual-energy x-ray absorptiometry analysis; HOMA IR-homeostatic model assessment for insulin resistance; OGTT-oral glucose tolerance test; TG-triglycerides; TC-total cholesterol; HDL-high density lipoprotein; LDL-low density lipoprotein; VLDL-very low density lipoprotein; ABPM-ambulatory blood preasure measurement; hsCRP-high sensitivity C-reactive protein; cIMT-carotid intima media thickness; ˜ for the studies in which the type of CCAH (SW/SV) in study group wasn’t specified in the paper, just the number of patients is presented in the table; ˚ unfavourable metabolic phenotype detected in children with CCAH compared to the control group or specific population regarding the specified parameter.

## Data Availability

No new data were created in this study. All analyzed studies and findings from studies were cited in the manuscript and are available online via citation search.
